# 

**Published:** 1921-02

**Authors:** 


### ANNOUNCEMENTS

### Alumni Society of Dewey School of Orthodontia

The next annual meeting of this society will be held on April 25th and 26th at the Hotel Ambassador in Atlantic City. The usual high standard of the meetings of this society will be maintained. Clinics and evening sessions will be included in the programme. All interested in orthodontia are cordially invited to attend these meetings. George F. Burke, Secy., 741-43 David Whitney Bldg., Detroit, Mich.

### Kentucky State Dental Association

The next annual meeting of the Kentucky State Dental Association will be held in Louisville, Ky., April 13-16, 1921. Seelbach Hotel as headquarters. A clinical program of unusual interest is being arranged. Address all correspondence to W. M. Randall, Secy., Louisville, Ky.

### Odontological Society of Western Pennsylvania

On April 12th and 13th the Odontological Society of Western Pennsylvania will hold its fortieth annual spring meeting at the William Penn Hotel, Pittsburgh, Pa.

### Tennessee State Dental Association

The fifty-fourth annual meeting of the Tennessee State Dental Association will be held at Nashville, Tenn., May 17, 18, 19, 20, 1921. J. J. Vaughn, D.D.S., Chairman Publicity Committee.

### Massachusetts Dental Society

The fifty-seventh annual meeting will be held in Boston, at the Hotel Somerset, May 4, 5 and 6, 1921. A cordial invitation is extended to all recognized members of state and national societies. W. Vernon Ryder, Secy., 175 Newbury St.

